# Hemoglobin-albumin-lymphocyte-platelet score and early neurological deterioration in acute ischemic stroke: a single-center retrospective cohort study

**DOI:** 10.3389/fneur.2026.1798807

**Published:** 2026-06-30

**Authors:** Ci Zhang, Wei Zhang, Yinyang Zhang, Guosong Xue

**Affiliations:** 1 Department of Neurology, Affiliated Hospital of Xuzhou Medical University, Xuzhou, Jiangsu, China; 2 Department of Neurology, Suqian Hospital of Nanjing Drum Tower Hospital Group, Suqian, Jiangsu, China; 3 School of Medical Imaging, Xuzhou Medical University, Xuzhou, Jiangsu, China

**Keywords:** acute ischemic stroke, clinical study, early neurological deterioration, hemoglobin-albumin-lymphocyte-platelet, nomogram

## Abstract

**Purpose:**

To investigate the association between the hemoglobin-albumin-lymphocyte-platelet (HALP) score and early neurological deterioration (END) in individuals with stroke and to develop an exploratory prediction model for END.

**Patients and methods:**

Clinical data from 595 patients with acute ischemic stroke (AIS) admitted to the Affiliated Hospital of Xuzhou Medical University from April 2022 to April 2024 were retrospectively analyzed. Patients were randomly divided into a training set and a validation set in a 7:3 ratio. Multivariable logistic regression analysis was utilized in the training data to examine END risk variables and build a corresponding predictive model. Model performance was evaluated using receiver operating characteristic (ROC) curves to assess discrimination and the Hosmer–Lemeshow goodness-of-fit test to evaluate calibration. Clinical decision curve analysis (DCA) was further applied to determine the model’s clinical value. To improve interpretability, the importance of included predictors was assessed using the SHapley Additive exPlanation (SHAP) method.

**Results:**

Of the 595 patients, 186 (31.3%) developed early neurological deterioration (END), while the remaining 409 (68.7%) comprised the non-END group. The training cohort consisted of 416 randomly assigned participants. Multivariate logistic regression showed that the large artery atherosclerosis (LAA) subtype, elevated baseline National Institutes of Health Stroke Scale (NIHSS) score, and lower HALP score constituted independent determinants for END development (*p* < 0.05). In further stepwise-adjusted analyses, the association between HALP and END remained statistically significant after controlling for demographic characteristics, vascular risk factors, and treatment-related variables. A nomogram incorporating HALP score, LAA subtype, and baseline NIHSS score showed moderate discrimination in both the training and validation sets.

**Conclusion:**

A lower HALP score was associated with END in patients with AIS. A nomogram incorporating HALP score, LAA subtype, and baseline NIHSS score demonstrated moderate discrimination and may provide useful information for early risk stratification in AIS. This prediction model requires validation in a larger, prospective clinical study.

## 1 Introduction

Acute ischemic stroke (AIS) incidence, death, disability, and recurrence rates have all steadily increased in recent years due to the aging population (1). The progressive worsening of neurological impairments within hours or days following the beginning of AIS is known as early neurological deterioration (END), a serious consequence of ischemic stroke. Although standardized diagnostic criteria or a uniform definition for END are lacking, multiple cohort studies have consistently associated END with adverse clinical outcomes (2–4).

The mechanistic basis of END likely involves two interdependent pathways: hemodynamic compromise and systemic metabolic derangement. Hemodynamic instability manifests as critical vascular disturbances—notably sustained arterial occlusion and inadequate collateral circulation—culminating in reduced cerebral perfusion pressure. Concurrently, metabolic dysregulation initiates complex neuropathological processes, principally characterized by ischemia-mediated neurotoxicity and hypoxia-induced inflammatory responses. These mechanisms act jointly to potentiate secondary brain injury (5–7). In addition, malnutrition, commonly observed in AIS patients, exacerbates complications and independently increases the risk of END, further worsening clinical outcomes (8). Previous studies have identified high NIHSS scores, large-artery atherosclerosis (LAA) subtype as classified by the Trial of Org 10,172 in Acute Stroke Treatment (TOAST), advanced age, and diabetes as factors associated with END (9–12). Elevated NIHSS scores and LAA subtype indicate significant blood flow impairment in large or functionally critical brain regions, which increases susceptibility to failed reperfusion, cerebral edema, hemorrhagic transformation, or infarct growth, thereby contributing to END. Some blood markers can reflect inflammatory status and offer clinical advantages due to their high accessibility and objectivity. Prior research has confirmed the prognostic significance of inflammatory and nutritional biomarkers (including neutrophil count, lymphocyte count, interleukin-6 [IL-6], and albumin levels) in predicting END (13, 14). However, the predictive capacity of these individual biomarkers is inherently limited due to inter-individual variability. Consequently, there is a pressing need to develop composite biomarkers for more comprehensive risk stratification (8).

The hemoglobin-albumin-lymphocyte-platelet (HALP) score is an integrated immunoinflammatory index that combines hemoglobin levels, lymphocyte counts, albumin levels, and platelet counts (15). This composite score integrates conventional biomarkers to evaluate patients across immunological, inflammatory, nutritional, and hematological dimensions. It provides a more holistic assessment of the equilibrium between immunological and inflammatory reactions. According to recent research, in AIS patients, a low HALP score correlates with elevated likelihood of poor prognosis, mortality, and post-stroke cognitive impairment. Other studies have suggested that thrombolysis status and NIHSS score at admission may be associated with END (16, 17). It is uncertain, therefore, if the HALP score and END are related (18). Hence, this study aimed to evaluate the association between HALP score and END in AIS patients. We further sought to establish and validate a nomogram incorporating additional factors to assist in END assessment.

## 2 Patients and methods

### 2.1 Patients

This study retrospectively included 595 eligible patients, consisting of 520 patients with AIS who received thrombolytic or conservative treatment in the general ward of the Department of Neurology at the Affiliated Hospital of Xuzhou Medical University, during a 2-year period from April 2022 to April 2024, and 75 patients who were transferred to the stroke ward after emergency thrombectomy during the same period. To effectively observe END, the study focused on all AIS patients. All patients received guideline-based stroke care for vascular risk factor control, including statins if eligible, to ensure consistent management of modifiable vascular risks. Inclusion criteria: (1) Patients met the AIS diagnostic criteria outlined in the *Chinese Stroke Association guidelines for clinical management of ischaemic cerebrovascular diseases: executive summary and 2023 update* (19); (2) Have been hospitalized within 72 h from symptom onset and have completed hematological examinations; (3) Have cranial magnetic resonance imaging (MRI) confirming the presence of a fresh infarct lesion; (4) Be aged ≥18 years. Exclusion criteria: (1) Have experienced stroke, cerebral hemorrhage, or other cerebrovascular diseases within the past 3 months; (2) Be complicated by malignant tumors, autoimmune diseases, or other severe organic diseases; (3) Fail to adhere regularly to antiplatelet aggregation therapy or lipid-regulating/plaque-stabilizing medications post-discharge; (4) Refuse follow-up or be lost to follow-up; (5) Have incomplete clinical documentation.

The Xuzhou Medical University Ethics Committee gave their approval to this project (No. XYFY2024-KL020).

### 2.2 Standardization of blood sample collection and detection

All blood parameters of the included patients were collected and tested per unified standards: blood samples were collected within 24 h of hospital admission, and all assays were performed using standardized equipment and reagents by the Clinical Laboratory Center of the Affiliated Hospital of Xuzhou Medical University, ensuring the comparability and reliability of blood biomarkers.

### 2.3 Data collection

Comprehensive patient data were collected, encompassing: demographics [gender, age, body mass index (BMI)], admission blood pressure [systolic blood pressure (SBP) and diastolic blood pressure (DBP)] measurements, cerebrovascular disease risk factors [hypertension, diabetes, coronary artery disease (CAD), atrial fibrillation (A-fib), dyslipidemia], National Institutes of Health Stroke Scale (NIHSS) scores at onset and peak, imaging data (cranial MRI, cerebral angiography), laboratory test results within 24 h of admission (lymphocyte count, platelet count, hemoglobin concentration, serum albumin level, etc.), receipt of thrombolytic therapy or endovascular thrombectomy, TOAST classification, and HALP score. HALP score is calculated using the following formula: hemoglobin (g/L) × albumin (g/L) × lymphocytes (×10^9^/L)/platelets (×10^9^/L) (15). For example, if a patient had a hemoglobin level of 140 g/L, an albumin level of 40 g/L, a lymphocyte count of 1.5 × 10^9^/L, and a platelet count of 200 × 10^9^/L, the HALP score would be 42.0. END lacks a universally accepted definition or standardized diagnostic criteria. In this study, we adopted a standard NIHSS-based definition of END for comparability with other studies, referring to the design of the Antiplatelet Therapy in Acute Minor Stroke and Transient Ischemic Attack (ATAMIS) randomized clinical trial. END was defined as an increase in the total NIHSS score of ≥2 points or an increase in the motor item score of 1 point within 7 days after symptom onset (20).

### 2.4 Statistical analysis

Statistical analysis was performed using SPSS 27.0, R software version 4.3.3, and Python. Measurement data were summarized as mean ± SD (*x_* ± SD) if normally distributed, and independent samples *t*-tests were applied for evaluation. Data violating normality assumptions were reported as median (IQR) with Mann–Whitney *U* tests used for group comparisons. Nominal variables were presented as frequency (%), and chi-squared tests were used. Variables identified as statistically significant in univariate analysis were incorporated into multivariate logistic regression to identify risk factors for END. Stepwise-adjusted stratified analysis was performed to evaluate the association between HALP and END. A nomogram was generated using the rms package in R (version 4.3.3). Nomogram performance was evaluated by means of receiver operating characteristic (ROC) curves, calibration plots, and decision curve analysis (DCA). ROC curve analysis was used to evaluate the discriminative ability of the nomogram for END, with the area under the curve (AUC) quantifying model performance. In the training set, the optimal cutoff value of HALP was determined by maximizing the Youden index, which is defined as sensitivity + specificity − 1. The optimal cut-off value was then applied to the validation set, and the corresponding sensitivity and specificity were calculated, with their 95% confidence intervals estimated using the bootstrap method with 5,000 resamples. The ROC analysis framework was pre-specified, and the optimal cut-off value was evaluated further in the validation set. Python was used for supplementary analyses, including bootstrap validation, ROC curve plotting, decision curve analysis, and SHAP-based model interpretation. We adopted the SHapley Additive exPlanation (SHAP) method to provide *post-hoc* explanations, enhancing model interpretability by quantifying feature contributions and ranking features by importance. The statistical significance was considered as *p* < 0.05 (two-tailed).

## 3 Results

### 3.1 Patient population

**Figure 1:**
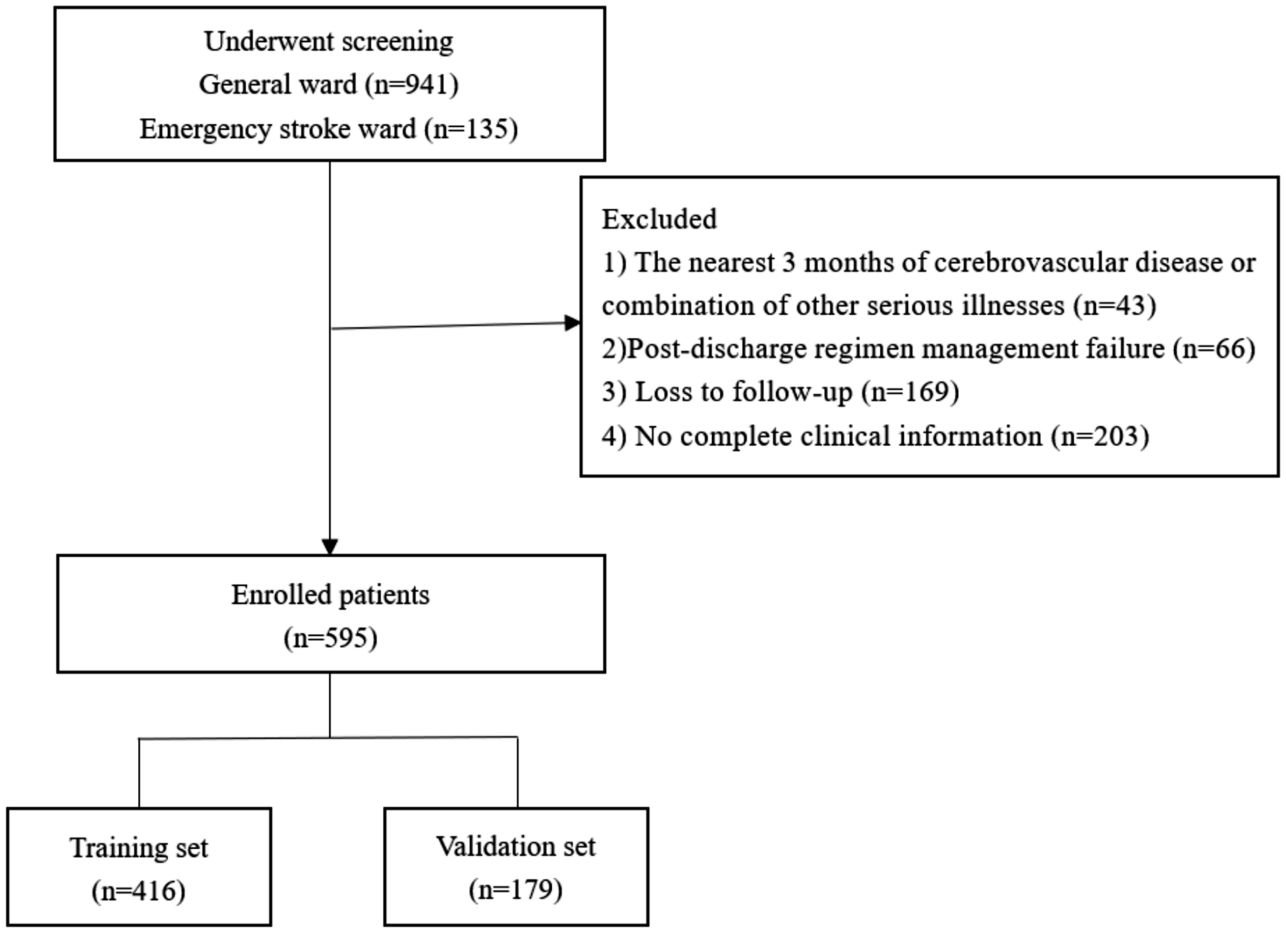
Study flowchart.

We enrolled 595 AIS patients, with 186 (31.26%) developing END. The patients were randomly divided into training and validation sets in an approximate 7:3 ratio, yielding 416 and 179 patients (Figure 1).

### 3.2 Baseline data comparison

Baseline characteristics were compared between the END and non-END groups within the training set. The END group exhibited higher values for NIHSS scores and a larger share of patients in the END group had LAA. In contrast, the END group had lower HALP scores, lymphocyte counts, hemoglobin levels, and albumin levels, along with a smaller proportion of patients receiving thrombolytic therapy. All comparisons yielded statistically significant differences (*p* < 0.05) (Table 1).

**Table 1:** Comparison of the general information of the END group and the non-END group in the training set.

Variables	Total (*n* = 416)	Non-END group (*n* = 286)	END group (*n* = 130)	Statistic	*p*
Age, years, mean ± SD	63.79 ± 11.74	63.43 ± 11.55	65.58 ± 12.16	*t* = −0.93	0.353
BMI, kg/m^2^, mean ± SD	25.32 ± 3.30	25.46 ± 3.37	25.01 ± 3.15	*t* = 1.28	0.202
SBP, mmHg, mean ± SD	149.12 ± 20.67	149.20 ± 21.74	148.95 ± 18.17	*t* = 0.12	0.908
DBP, mmHg, mean ± SD	87.38 ± 13.46	86.86 ± 13.56	88.52 ± 13.23	*t* = −1.17	0.243
Lymphocyte, ×10^9^/L, mean ± SD	1.67 ± 0.70	1.78 ± 0.75	1.42 ± 0.51	*t* = 5.09	<0.001
Hemoglobin, g/L, mean ± SD	141.42 ± 16.52	143.24 ± 15.77	137.39 ± 17.45	*t* = 3.39	<0.001
Platelet, ×10^9^/L, mean ± SD	216.32 ± 67.26	214.12 ± 68.48	221.18 ± 64.49	*t* = −0.99	0.322
Albumin, g/L, mean ± SD	41.09 ± 3.77	41.45 ± 3.74	40.30 ± 3.74	*t* = 2.92	0.004
NIHSS, median (IQR)	4.00 (3.00–6.00)	3.00 (2.00–5.00)	4.00 (3.00–5.50)	*Z* = -6.77	<0.001
HALP, median (IQR)	43.74 (32.51–59.72)	47.88 (36.31–63.85)	35.20 (26.95–48.31)	*Z* = -6.27	<0.001
SCR, μmol/L, median (IQR)	59.00 (50.25–69.75)	60.00 (50.00–70.00)	58.50 (50.75–68.25)	*Z* = -0.53	0.599
Male, *n* (%)	307 (73.8)	214 (74.83)	93 (71.54)	*χ*^2^ = 2.07	0.355
Hypertension, *n* (%)	270 (64.90)	184 (64.34)	86 (66.15)	*χ*^2^ = 1.30	0.719
Diabetes, *n* (%)	140 (33.65)	89 (31.12)	51 (39.23)	*χ*^2^ = 2.63	0.105
CAD, *n* (%)	66 (15.87)	47 (16.43)	19 (14.62)	*χ*^2^ = 0.22	0.638
Smoking, *n* (%)	133 (31.97)	95 (33.22)	38 (29.23)	*χ*^2^ = 0.65	0.419
Dyslipidemia, *n* (%)	53 (12.74)	41 (14.34)	12 (9.23)	*χ*^2^ = 2.10	0.148
LAA, *n* (%)	241 (57.93)	134 (46.85)	107 (82.31)	*χ*^2^ = 46.10	<0.001
A-fib, *n* (%)	33 (7.93)	23 (8.04)	10 (7.69)	*χ*^2^ = 0.02	0.903
Thrombolysis, *n* (%)	15 (3.61)	14 (4.90)	1 (0.77)	*χ*^2^ = 4.38	0.036
Thrombectomy, *n* (%)	56 (13.46)	39 (13.64)	17 (13.08)	*χ*^2^ = 0.024	0.877

END, early neurological deterioration; BMI, body mass index; SBP, systolic blood pressure; DBP, diastolic blood pressure; NIHSS, National Institutes of Health Stroke Scale; HALP, hemoglobin-albumin-lymphocyte-platelet; SCR, serum creatinine; CAD, coronary artery disease; LAA, large-artery atherosclerosis; A-fib, atrial fibrillation.

### 3.3 Univariate logistic regression analysis

The results revealed that: TOAST classification of LAA (OR = 5.28, 95%CI = 3.18–8.76, *p* < 0.01), a higher NIHSS score at admission (OR = 1.05, 95%CI = 1.02–1.08, *p* < 0.01), and a lower HALP score (OR = 0.96, 95%CI = 0.95–0.97, *p* < 0.01) were associated with END (Table 2).

**Table 2:** Univariate logistic regression analysis of END risk factors.

Variables	*β*	S.E	*Z*	*p*	OR (95%CI)
Age	0.01	0.01	0.86	0.353	1.01 (0.99–1.03)
BMI	−0.04	0.03	−1.28	0.202	0.96 (0.90–1.02)
SBP	−0.00	0.01	−0.12	0.908	1.00 (0.99–1.01)
DBP	0.01	0.01	1.17	0.243	1.01 (0.99–1.02)
NIHSS	0.05	0.02	3.13	0.002	1.05 (1.02–1.08)
HALP	−0.04	0.01	−5.88	<0.001	0.96 (0.95–0.97)
SCR	0.00	0.01	0.47	0.637	1.00 (0.99–1.01)
Male	0.55	0.66	0.84	0.400	1.74 (0.48–6.30)
Hypertension	0.08	0.22	0.36	0.719	1.08 (0.70–1.68)
Diabetes	0.36	0.22	1.62	0.105	1.43 (0.93–2.20)
CAD	−0.14	0.30	−0.47	0.638	0.87 (0.49–1.55)
LAA	1.66	0.26	6.43	<0.001	5.28 (3.18–8.76)
Smoking	−0.19	0.23	−0.81	0.419	0.83 (0.53–1.30)
Dyslipidemia	−0.50	0.35	−1.44	0.151	0.61 (0.31–1.20)
A-fib	−0.05	0.39	−0.12	0.903	0.95 (0.44–2.06)
Thrombolysis	−1.89	1.04	−1.82	0.069	0.15 (0.02–1.16)
Thrombectomy	−0.05	0.31	−0.15	0.877	0.95 (0.52–1.76)

OR, odds ratio; CI, confidence interval; BMI, body mass index; SBP, systolic blood pressure; DBP, diastolic blood pressure; NIHSS, National Institutes of Health Stroke Scale; HALP, hemoglobin-albumin-lymphocyte-platelet; SCR, Serum Creatinine; CAD, coronary artery disease; LAA, large-artery atherosclerosis; A-fib, atrial fibrillation.

### 3.4 Multivariable logistic regression analysis

Given that the HALP score is composed of four components (lymphocyte count, hemoglobin level, platelet count, and albumin level), and to avoid potential inaccuracies in the results due to multicollinearity, these four individual components were excluded from the analysis. NIHSS score, HALP score, and TOAST categorization of LAA were among the variables shown to be substantially linked with END by univariate logistic regression analysis (*p* < 0.05). Following this selection, they were subjected to a multivariate logistic regression analysis. The TOAST classification of LAA (OR = 4.85, 95%CI = 2.86–8.23, *p* < 0.01), a higher NIHSS score at admission (OR = 1.04, 95%CI = 1.01–1.08, *p* < 0.05), and a lower HALP score (OR = 0.96, 95%CI = 0.95–0.98, *p* < 0.01) were independent risk factors for END (Table 3).

**Table 3:** Multivariate logistic regression analysis of END risk factors.

Variables	*β*	S.E	*Z*	*p*	OR (95%CI)
Intercept	−0.48	0.40	−1.19	0.233	0.62 (0.28–1.36)
LAA	1.58	0.27	5.86	<0.001	4.85 (2.86–8.23)
NIHSS	0.04	0.02	2.30	0.021	1.04 (1.01–1.08)
HALP	−0.04	0.01	−5.10	<0.001	0.96 (0.95–0.98)

OR, odds ratio; CI, confidence interval; LAA, large-artery atherosclerosis; NIHSS, National Institutes of Health Stroke Scale; HALP, hemoglobin-albumin-lymphocyte-platelet.

### 3.5 Stepwise-adjusted associations of HALP with END in AIS patients

In the training set, lower HALP demonstrated a significant inverse association with END risk in the unadjusted model (Model 1: OR = 0.96, 95%CI: 0.95–0.97, *p* < 0.01). This association persisted after sequential adjustment for demographic factors and smoking status (Model 2: OR = 0.96, 95%CI: 0.95–0.97, *p* < 0.01), vascular comorbidities (Model 3: OR = 0.96, 95%CI: 0.95–0.97, *p* < 0.01), and A-fib along with treatment-related variables (Model 4: OR = 0.96, 95%CI: 0.94–0.97, *p* < 0.01), the association between HALP and END remained robust in the fully adjusted model (Table 4).

**Table 4:** Stepwise-adjusted associations of HALP with END in training cohort.

Variables	Model 1			Model 2			Model 3			Model 4		
	*β*	OR (95%CI)	*p*-value	*β*	OR (95%CI)	*p*-value	*β*	OR (95%CI)	*p*-value	*β*	OR (95%CI)	*p*-value
HALP	−0.04	0.96 (0.95–0.97)	4.184 × 10^−9^	−0.04	0.96 (0.95–0.97)	1.097 × 10^−8^	−0.04	0.96 (0.95–0.97)	8.126 × 10^−9^	−0.04	0.96 (0.94–0.97)	5.971 × 10^−9^
Age	—	—	—	−3.00 × 10^−3^	1.00 (0.98–1.02)	0.792	−1.00 × 10^−3^	1.00 (0.98–1.02)	0.928	−2.00 × 10^−3^	1.00 (0.98–1.02)	0.863
Sex	—	—	—	−0.07	0.93 (0.57–1.53)	0.777	−0.13	0.88 (0.53–1.45)	0.606	−0.15	0.87 (0.52–1.44)	0.578
BMI	—	—	—	−0.02	0.98 (0.92–1.05)	0.604	−0.02	0.98 (0.92–1.05)	0.583	−0.01	0.99 (0.92–1.06)	0.690
Smoking	—	—	—	−0.20	0.82 (0.51–1.32)	0.410	−0.23	0.80 (0.49–1.29)	0.355	−0.28	0.76 (0.47–1.23)	0.265
Hypertension	—	—	—	—	—	—	0.21	1.24 (0.76–2.01)	0.396	0.24	1.27 (0.77–2.09)	0.345
Diabetes	—	—	—	—	—	—	0.45	1.57 (0.98–2.53)	0.062	0.44	1.55 (0.96–2.50)	0.075
CAD	—	—	—	—	—	—	−0.23	0.80 (0.42–1.49)	0.475	−0.30	0.74 (0.38–1.45)	0.384
Dyslipidemia	—	—	—	—	—	—	−0.41	0.66 (0.32–1.38)	0.271	−0.38	0.68 (0.33–1.43)	0.313
A-fib	—	—	—	—	—	—	—	—	—	0.21	1.23 (0.49–3.13)	0.658
Thrombectomy	—	—	—	—	—	—	—	—	—	−0.22	0.80 (0.39–1.65)	0.548
Thrombolysis	—	—	—	—	—	—	—	—	—	−2.03	0.13 (0.02–1.05)	0.055

Model 1 was unadjusted and included HALP only. Model 2 was adjusted for age, sex, BMI, and smoking. Model 3 was further adjusted for hypertension, diabetes, CAD, and dyslipidemia. Model 4 was additionally adjusted for A-fib, thrombectomy, and thrombolysis. OR, odds ratio; CI, confidence interval; HALP, hemoglobin-albumin-lymphocyte-platelet; BMI, body mass index; CAD, coronary artery disease; A-fib, atrial fibrillation.

In the validation set, the direction of association between HALP and END remained consistent, and the association retained statistical significance (Supplementary Table 1). To further determine whether different treatment modalities affect the association between HALP and END, we conducted heterogeneity analyses with stepwise adjustment for potential confounders. The results demonstrated a persistent association between HALP and END across treatment subgroups (Supplementary Tables 2–5).

### 3.6 Development and validation of a nomogram

**Figure 2:**
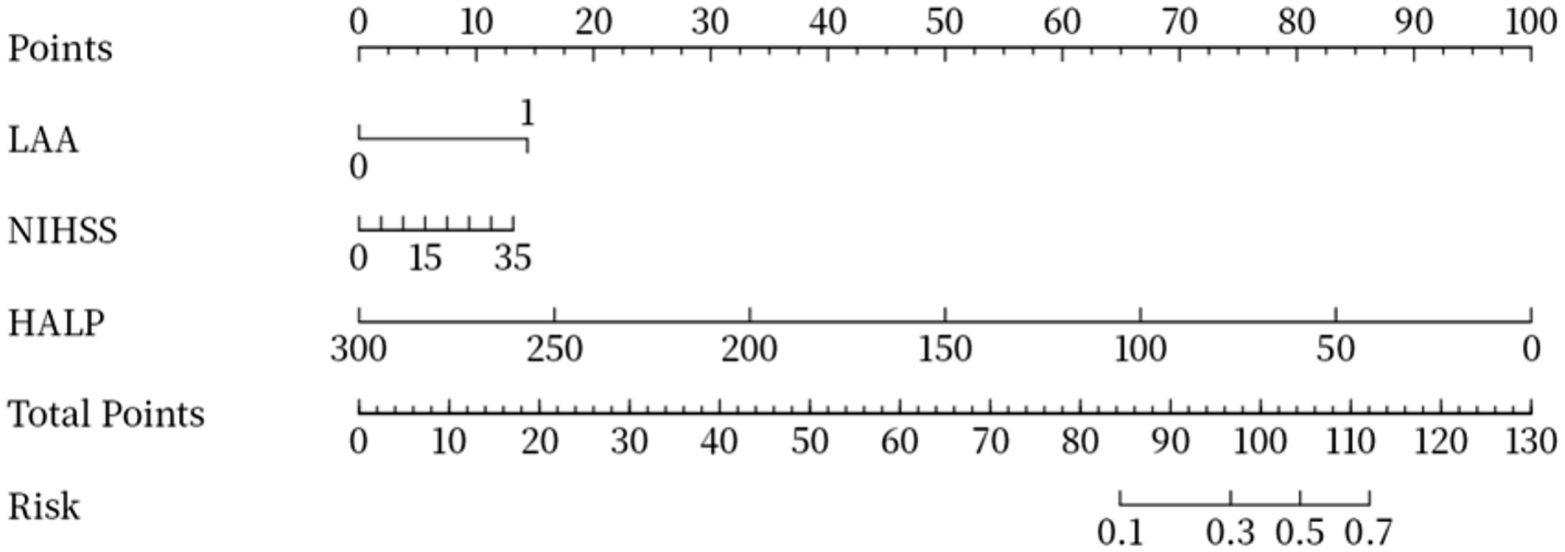
Nomogram model for predicting END in patients with AIS. END, early neurological deterioration; AIS, acute ischemic stroke; LAA, large-artery atherosclerosis; NIHSS, National Institutes of Health Stroke Scale; HALP, hemoglobin-albumin-lymphocyte-platelet.

**Figure 3:**
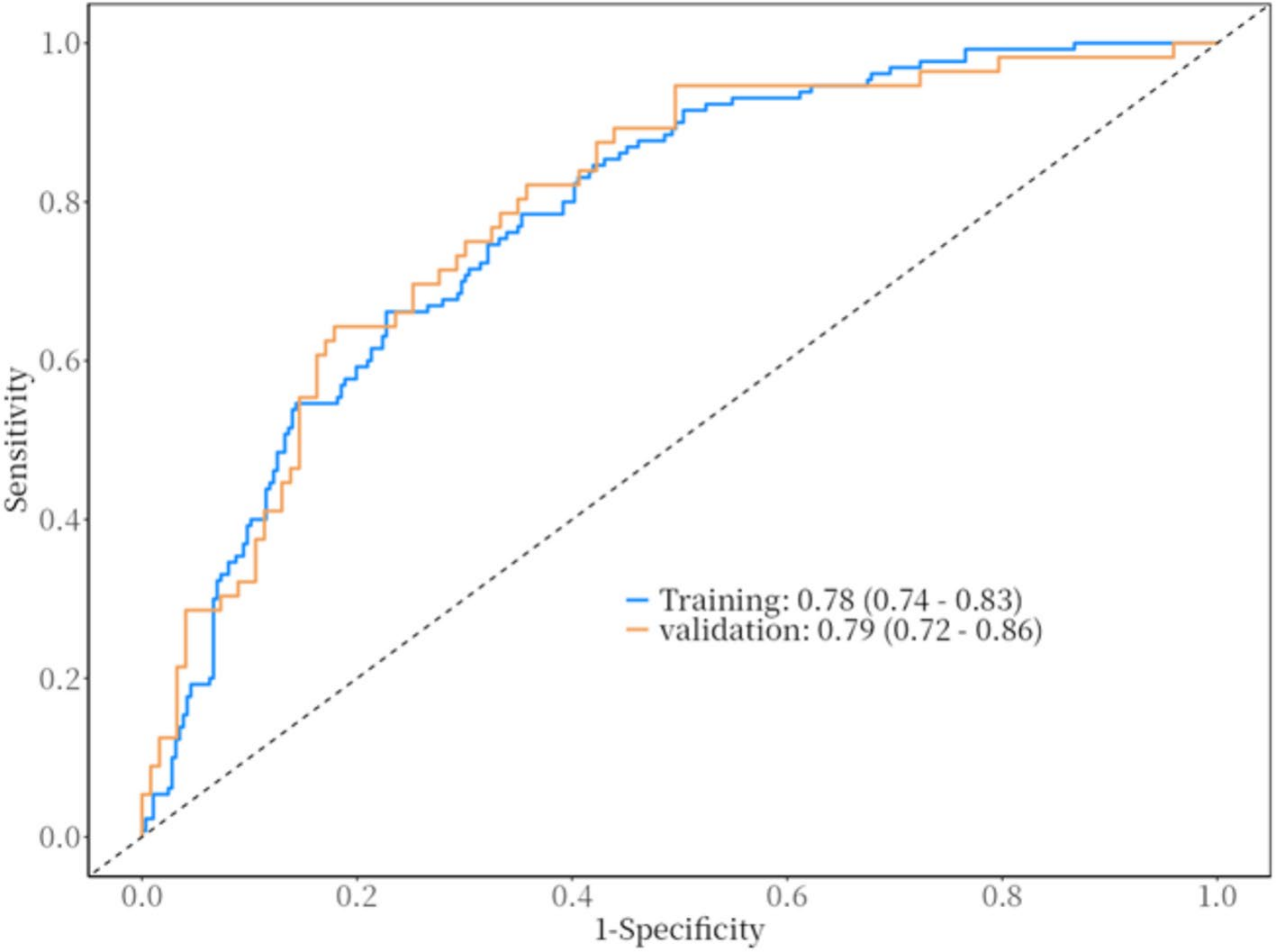
ROC curve of the nomogram model for predicting END risk. ROC, receiver operating characteristic; END, early neurological deterioration.

**Figure 4:**
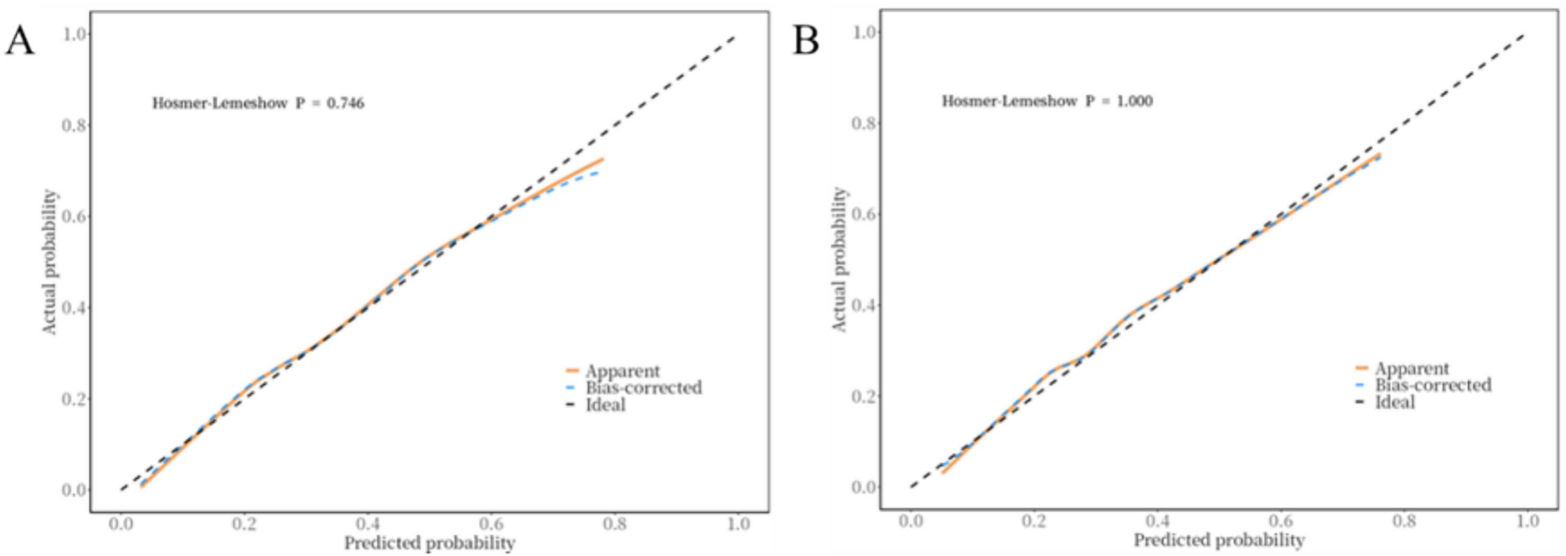
The calibration curve of the nomogram model for END risk in AIS patients. **(A)** Training set, **(B)** validation set. END, early neurological deterioration; AIS, acute ischemic stroke.

**Figure 5:**
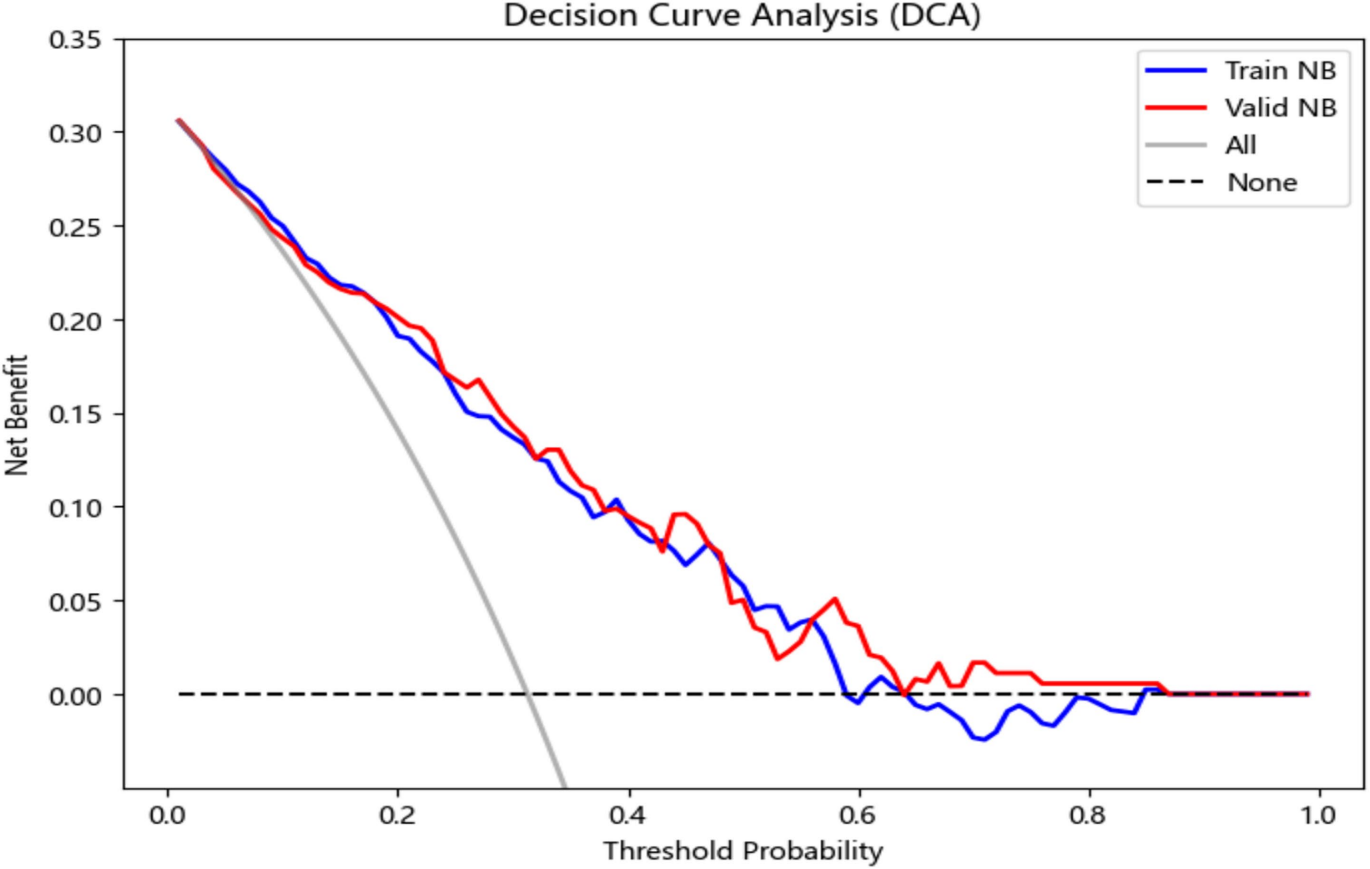
The DCA curve of the nomogram model for END risk in AIS patients. END, early neurological deterioration; AIS, acute ischemic stroke; DCA, decision curve analysis.

**Figure 6:**
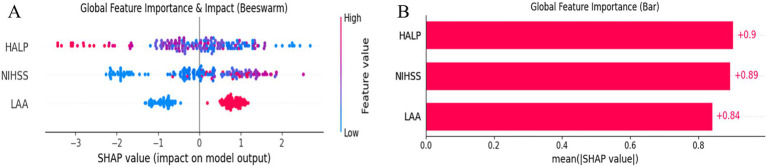
SHAP global interpretation of the END prediction model. **(A)** SHAP Beeswarm: One feature per line. Each point represents the SHAP value for an individual observation of that feature. The horizontal axis represents SHAP values: >0 indicates a positive contribution to increased predicted risk, <0 indicates reduced risk. The color gradient from blue to red represents low to high actual feature values, respectively. **(B)** Bar chart of features sorted by mean |SHAP| value: Feature importance rankings are HALP, NIHSS, and LAA. SHAP, SHapley Additive explanation; END, early neurological deterioration; HALP, hemoglobin-albumin-lymphocyte-platelet; NIHSS, National Institutes of Health Stroke Scale; LAA, large-artery atherosclerosis.

**Figure 7:**
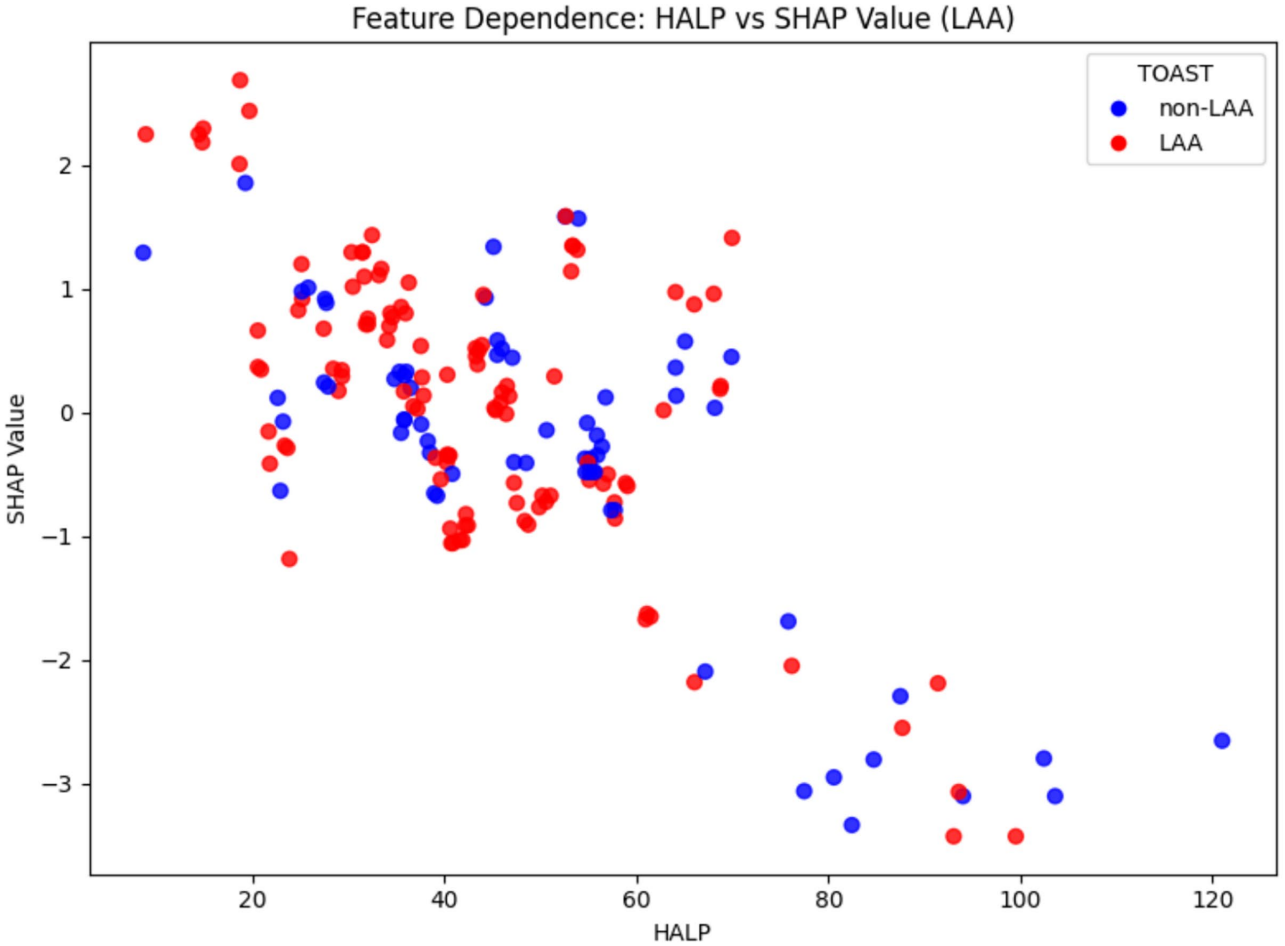
SHAP dependence plot for HALP in the END prediction model. SHAP dependence plot depicting the relationship between HALP levels and corresponding SHAP values, colored by TOAST subtype (LAA vs. non-LAA). SHAP, SHapley Additive explanation; END, early neurological deterioration; TOAST, Trial of Org 10,172 in Acute Stroke Treatment; LAA, large-artery atherosclerosis.

A nomogram was constructed to predicted the risk of END, incorporating three predictors: TOAST classification of LAA, admission NIHSS score, and HALP score (Figure 2). The predictive performance of the nomogram was assessed using ROC curve analysis. The area under the curve (AUC) was 0.78, with a sensitivity of 66% and a specificity of 77%, indicating its moderate discrimination. In the validation set, the AUC was 0.79, suggesting similar moderate discriminative performance (Figure 3). Calibration was examined using the Hosmer-Lemeshow test and calibration curves. The calibration curves demonstrated generally acceptable concordance between the predicted and observed probabilities (Figure 4). The nomogram’s clinical usefulness was evaluated using DCA. Decision curve analysis showed that the nomogram yielded a positive net benefit across a clinically relevant range of threshold probabilities, with the net benefit remaining positive until approximately 59.0% in the training set and 64.0% in the validation set (Figure 5). SHAP analysis indicated the relative contributions of HALP, NIHSS, and LAA on predicted risk. The SHAP swarm plot revealed variations in magnitude and direction of feature effects. Overall, elevated NIHSS and LAA subtype predicted higher risk, whereas increased HALP levels correlated with reduced risk (Figure 6). The SHAP dependence plot for HALP indicated an inverse relationship: with increasing HALP levels, corresponding SHAP values decreased, suggesting higher HALP may indicate lower risk in AIS patients (Figure 7).

## 4 Discussion

Our findings showed that reduced HALP scores were significantly associated with END, and this association remained stable after adjustment for demographic characteristics, vascular risk factors, and treatment-related variables. Additionally, we developed a clinical nomogram incorporating HALP score, LAA subtype, and baseline NIHSS score. The model demonstrated consistent moderate discrimination in both training and validation sets, suggesting that HALP may provide supplementary information beyond its association for early risk stratification in AIS patients. During AIS, the immune system initiates a sterile inflammatory response, with inflammatory factor accumulation and inflammatory cascades at the ischemic lesion site synergistically contributing to END development (21).

The HALP score is calculated based on four routinely available laboratory parameters—hemoglobin, albumin, lymphocytes, and platelets—and has the advantages of accessibility, low cost, and good reproducibility (15). From a biological perspective, HALP demonstrates biologically plausible relevance as a biomarker associated with END. Lymphocytes serve as critical regulators of the post-ischemic immune response in acute ischemic stroke. Lymphopenia may indicate stroke-induced immunosuppression and is associated with increased infection risk, impaired tissue repair, and poorer neurological outcomes; however, some lymphocyte subsets may exert neuroprotective immunomodulatory effects after ischemic injury (22). Platelets are key mediators of both thrombosis and thrombo-inflammatory activity. Platelet activation may aggravate microcirculatory dysfunction, endothelial injury, and blood–brain barrier disruption, contributing to infarct progression and neurological worsening (23). This thrombo-inflammatory cascade may partially explain why higher platelet count in the HALP formula correlates with END risk. Hemoglobin serves as an indicator of nutritional status and demonstrates a U-shaped relationship with AIS prognosis, where both hypohemoglobinemia and hyperhemoglobinemia are associated with poor outcomes (24). Low hemoglobin levels increase infarct size and compromise cerebrovascular autoregulation, worsening ischemia and hypoxia in the ischemic penumbra (25–27). Furthermore, low hemoglobin induces cerebral blood flow turbulence, promoting thrombus migration and upregulating vascular endothelial adhesion factor expression, thus triggering inflammatory responses and thrombosis. It may further upregulate inflammatory mediators (e.g., nitric oxide synthase and C-X-C chemokine receptors), collectively aggravating brain damage (28). Serum albumin reflects nutritional status and is closely associated with prognosis in AIS. As an anti-inflammatory and antioxidant molecule, albumin exerts neuroprotective effects by attenuating oxidative stress, reducing cerebral edema, and inhibiting platelet aggregation. Lower albumin levels are associated with poor functional recovery, increased infection risk, and unfavorable prognosis in AIS patients (29, 30). Accordingly, HALP may simultaneously capture several interrelated biological processes after stroke, including immune dysregulation, enhanced thrombo-inflammation, impaired oxygen delivery, and reduced nutritional and antioxidant reserves. A lower HALP score therefore reflects a state of increased inflammatory burden and reduced physiological reserve, which may render patients more vulnerable to neurological worsening. Based on previous evidence, reduced HALP scores independently predict adverse outcomes in patients with AIS (31). Our findings extend these observations to END, an acute-phase complication associated with clinically significant outcomes, which may explain the mechanism underlying poor prognosis.

Thrombolysis and thrombectomy are critical interventional measures for acute occlusive stroke (32, 33). They salvage the ischemic penumbra by achieving vascular recanalization and restoring blood perfusion. Currently, the association between thrombolysis or thrombectomy and END remains unclear, and our study failed to show a significant correlation. This may be attributed to the fact that END is significantly influenced by multiple factors. Even with successful vascular recanalization, END may still occur if downstream microcirculatory failure prevents effective perfusion of the brain tissue. Restoration of blood flow may trigger inflammatory and oxidative stress responses, which instead exacerbate cerebral edema and hemorrhagic transformation, directly leading to END (34, 35).

Considering that patients undergoing conservative treatment, intravenous thrombolysis, and mechanical thrombectomy may differ in baseline stroke severity, vascular status, monitoring intensity, and the risk of neurological deterioration after treatment, and that these differences may in turn affect the risk of END, we used stepwise adjustment to exclude the influence of confounding factors. The results suggested that the association between HALP and END was robust across treatment modalities.

This study also identified the TOAST subtype of LAA and higher admission NIHSS scores as independent risk factors for END. These findings are consistent with earlier reports (36). Higher NIHSS scores reflect greater stroke severity, larger infarct volumes, and higher chances of sequelae such hemorrhagic transformation and cerebral edema. These factors collectively elevate the risk of END (37). LAA-related infarction increases END risk due to extensive vascular lesions, impaired blood flow regulation, and insufficient post-stroke perfusion (38). Based on these identified risk factors, we constructed an exploratory integrated model for END in AIS patients. The model exhibited moderate discrimination in both the training and validation sets, suggesting that incorporating HALP into established clinical risk factors may help enable earlier risk stratification in AIS. Close monitoring is recommended for patients with an elevated nomogram-derived risk score at admission. More intensive therapeutic interventions could potentially reduce the incidence of END.

This research still has several limitations. First, the retrospective single-center design limits causal interpretation and the generalizability of the findings. In addition, although random split-sample validation and bootstrap-based internal validation were performed, these approaches cannot substitute for external validation in an independent cohort. Therefore, all model-related findings should be regarded as exploratory. Second, excluding patients with incomplete follow-up, missing data, or poor post-discharge adherence may have disproportionately removed high-risk individuals, introducing selection bias. Third, END was defined using a commonly adopted NIHSS-based criterion, but divergent definitions in previous studies may limit cross-study comparability. Fourth, although treatment-related variables (e.g., intravenous thrombolysis and mechanical thrombectomy) were included in additional adjusted analyses, residual confounding and treatment heterogeneity cannot be completely excluded; thus, the observed associations require cautious interpretation. Finally, HALP was measured at a single hospitalization time point, precluding assessment of dynamic changes. Future studies should explore the predictive value of serial HALP measurements. Further validation of this model against established models in large prospective multicenter cohorts is essential for refinement.

## 5 Conclusion

A lower HALP score was associated with END in patients with AIS. A nomogram incorporating HALP score, LAA subtype, and baseline NIHSS score demonstrated moderate discrimination and may provide useful information for early risk stratification in AIS. This prediction model requires validation in a larger, prospective clinical study.

## Data Availability

The raw data supporting the conclusions of this article will be made available by the authors, without undue reservation.

## Glossary

A-fib: atrial fibrillation
AIS: acute ischemic stroke
ATAMIS: Antiplatelet Therapy in Acute Minor Stroke and Transient Ischemic Attack
AUC: area under the curve
BMI: body mass index
CAD: coronary artery disease
CI: confidence interval
DBP: diastolic blood pressure
DCA: decision curve analysis
END: early neurological deterioration
HALP: hemoglobin-albumin-lymphocyte-platelet
IL-6: interleukin-6
IQR: interquartile range
LAA: large-artery atherosclerosis
MRI: magnetic resonance imaging
NIHSS: National Institutes of Health Stroke Scale
OR: odds ratio
ROC: receiver operating characteristic
SBP: systolic blood pressure
SCR: serum creatinine
SD: standard deviation
SHAP: SHapley Additive exPlanation
SPSS: Statistical Package for the Social Sciences
TOAST: Trial of Org 10,172 in Acute Stroke Treatment
