# Supplementary material for: Hemoglobin-albumin-lymphocyte-platelet score and early neurological deterioration in acute ischemic stroke: a single-center retrospective cohort study
Source: Front Neurol. 2026 Jun 30;17:1798807. doi: 10.3389/fneur.2026.1798807 (PMC13364552; doi:10.3389/fneur.2026.1798807)
Supplement: Supplementary file 1 [file Data_Sheet_1.PDF]

Supplementary Table 1. Stepwise-adjusted associations of HALP with END in the validation cohort

Variables	Model 1			Model 2			Model 3			Model 4		
	**β**	**OR（95%CI）**	***P***	**β**	**OR（95%CI）**	***P***	**β**	**OR（95%CI）**	***P***	**β**	**OR（95%CI）**	***P***
HALP	-0.05	0.96 (0.93–0.978)	7.102×10^{-5}	-0.04	0.96 (0.94–0.98)	4.174×10^{-4}	-0.05	0.96 (0.93–0.98)	2.591×10^{-4}	-0.05	0.96 (0.93–0.98)	2.362×10^{-4}
Age	—	—	—	0.02	1.02 (0.99–1.05)	0.243	0.02	1.02 (0.99–1.05)	0.311	0.02	1.02 (0.99–1.05)	0.268
Sex	—	—	—	-0.45	0.64 (0.29–1.42)	0.272	-0.65	0.52 (0.22–1.22)	0.132	-0.62	0.54 (0.22–1.29)	0.164
BMI	—	—	—	-0.09	0.91 (0.82–1.02)	0.106	-0.13	0.88 (0.77–0.99)	0.034	-0.13	0.88 (0.78–0.99)	0.041
Smoking	—	—	—	-0.48	0.62 (0.26–1.49)	0.284	-0.61	0.54 (0.22–1.36)	0.192	-0.69	0.50 (0.20–1.26)	0.144
Hypertension	—	—	—	—	—	—	0.42	1.53 (0.65–3.61)	0.336	0.43	1.54 (0.63–3.74)	0.345
Diabetes	—	—	—	—	—	—	0.55	1.73 (0.81–3.71)	0.160	0.47	1.60 (0.73–3.51)	0.241
CAD	—	—	—	—	—	—	-0.29	0.75 (0.29–1.94)	0.552	-0.16	0.85 (0.32–2.31)	0.755
Dyslipidemia	—	—	—	—	—	—	1.15	3.14 (1.12–8.81)	0.030	0.95	2.59 (0.90–7.42)	0.076
A-fib	—	—	—	—	—	—	—	—	—	-0.75	0.472(0.09–2.53)	0.381
Thrombectomy	—	—	—	—	—	—	—	—	—	-0.42	0.66 (0.14–2.99)	0.585
Thrombolysis	—	—	—	—	—	—	—	—	—	-1.42	0.24 (0.05–1.22)	0.085

Note: Model 1 was unadjusted and included HALP only. Model 2 was adjusted for age, sex, BMI, and smoking. Model 3 was further adjusted for hypertension, diabetes, CAD, and dyslipidemia. Model 4 was additionally adjusted for A-fib, thrombectomy, and thrombolysis. OR, odds ratio; CI, confidence interval; HALP, hemoglobin-albumin-lymphocyte-platelet; BMI, body mass index; CAD, coronary artery disease; A-fib, atrial fibrillation.

Supplementary Table 2. Heterogeneity analysis in the non-thrombolysis training cohort

Variables	Model 1			Model 2			Model 3			Model 4		
	**β**	**OR（95%CI）**	***P***	**β**	**OR（95%CI）**	***P***	**β**	**OR（95%CI）**	***P***	**β**	**OR（95%CI）**	***P***
HALP	-0.04	0.96 (0.95–0.97)	7.822×10^{-9}	-0.04	0.96 (0.95–0.97)	1.978×10^{-8}	-0.041	0.96 (0.95–0.97)	1.355×10^{-8}	-0.04	0.96 (0.95–0.97)	1.314×10^{-8}
Age	—	—	—	-1.00×10^{-3}	1.00 (0.98–1.02)	0.913	1.00×10^{-3}	1.00 (0.98–1.02)	0.939	$<1.00 \times 10^{-3}$	1.00 (0.98–1.02)	0.980

Sex	—	—	—	-0.12	0.89 (0.54–1.47)	0.647	-0.19	0.83 (0.49–1.38)	0.469	-0.19	0.83 (0.49–1.39)	0.473
BMI	—	—	—	-0.01	0.99 (0.92–1.06)	0.683	-0.02	0.98 (0.92–1.06)	0.649	-0.02	0.98 (0.92–1.06)	0.649
Smoking	—	—	—	-0.23	0.79 (0.49–1.28)	0.343	-0.26	0.77 (0.48–1.26)	0.301	-0.27	0.77 (0.47–1.25)	0.282
Hypertension	—	—	—	—	—	—	0.24	1.27 (0.77–2.07)	0.347	0.23	1.25 (0.76–2.06)	0.374
Diabetes	—	—	—	—	—	—	0.45	1.56 (0.97–2.52)	0.069	0.45	1.57 (0.97–2.54)	0.065
CAD	—	—	—	—	—	—	-0.25	0.78 (0.41–1.47)	0.440	-0.30	0.74 (0.38–1.45)	0.386
Dyslipidemia	—	—	—	—	—	—	-0.37	0.69 (0.33–1.44)	0.323	-0.37	0.69 (0.33–1.46)	0.333
A-fib	—	—	—	—	—	—	—	—	—	0.19	1.21 (0.48–3.07)	0.684
Thrombectomy	—	—	—	—	—	—	—	—	—	-0.21	0.81 (0.39–1.67)	0.565

Note: Model 1 was unadjusted and included HALP only. Model 2 was adjusted for age, sex, BMI, and smoking. Model 3 was further adjusted for hypertension, diabetes, CAD, and dyslipidemia. Model 4 was additionally adjusted for A-fib and thrombectomy. OR, odds ratio; CI, confidence interval; HALP, hemoglobin-albumin-lymphocyte-platelet; BMI, body mass index; CAD, coronary artery disease; A-fib, atrial fibrillation.

Supplementary Table 3. Heterogeneity analysis in the non-thrombolysis validation cohort

Variables	Model 1			Model 2			Model 3			Model 4		
	β	OR（95%CI）	*P*	β	OR（95%CI）	*P*	β	OR（95%CI）	*P*	β	OR（95%CI）	*P*
HALP	-0.05	0.96 (0.93–0.98)	7.545×10^{-5}	-0.04	0.96 (0.94–0.98)	4.227×10^{-4}	-0.05	0.95 (0.93–0.98)	2.754×10^{-4}	-0.05	0.95 (0.93–0.98)	2.638×10^{-4}
Age	—	—	—	0.03	1.03 (1.00–1.06)	0.87	0.03	1.03 (1.00–1.06)	0.101	0.03	1.03 (1.00–1.07)	0.081
Sex	—	—	—	-0.48	0.62 (0.27–1.44)	0.267	-0.72	0.49 (0.20–1.21)	0.120	-0.68	0.51 (0.20–1.27)	0.148
BMI	—	—	—	-0.08	0.92 (0.82–1.03)	0.151	-0.13	0.88 (0.78–1.00)	0.051	-0.13	0.88 (0.78–1.00)	0.055
Smoking	—	—	—	-0.60	0.55 (0.23–1.33)	0.18	-0.74	0.48 (0.19–1.22)	0.121	-0.71	0.49 (0.19–1.26)	0.138
Hypertension	—	—	—	—	—	—	0.46	1.59 (0.64–3.97)	0.321	0.51	1.66 (0.65–4.23)	0.288
Diabetes	—	—	—	—	—	—	0.69	1.98 (0.89–4.42)	0.093	0.58	1.79 (0.79–4.05)	0.161
CAD	—	—	—	—	—	—	-0.49	0.62 (0.22–1.70)	0.350	-0.35	0.71 (0.25–2.02)	0.516
Dyslipidemia	—	—	—	—	—	—	1.09	2.96 (1.03–8.50)	0.043	0.98	2.67 (0.92–7.76)	0.071

A-fib	—	—	—	—	—	—	—	—	—	-0.85	0.43 (0.08–2.34)	0.326
Thrombectomy	—	—	—	—	—	—	—	—	—	-0.33	0.72 (0.15–3.35)	0.675

Note: Model 1 was unadjusted and included HALP only. Model 2 was adjusted for age, sex, BMI, and smoking. Model 3 was further adjusted for hypertension, diabetes, CAD, and dyslipidemia. Model 4 was additionally adjusted for A-fib and thrombectomy. OR, odds ratio; CI, confidence interval; HALP, hemoglobin-albumin-lymphocyte-platelet; BMI, body mass index; CAD, coronary artery disease; A-fib, atrial fibrillation.

Supplementary Table 4. Heterogeneity analysis in the non-thrombectomy training cohort

Variables	Model 1			Model 2			Model 3			Model 4		
	**β**	**OR（95%CI）**	***P***	**β**	**OR（95%CI）**	***P***	**β**	**OR（95%CI）**	***P***	**β**	**OR（95%CI）**	***P***
HALP	-0.06	0.94 (0.93–0.96)	4.356×10^{-11}	-0.06	0.94 (0.92–0.96)	6.084×10^{-11}	-0.06	0.94 (0.92–0.96)	5.549×10^{-11}	-0.06	0.94 (0.92–0.96)	3.673×10^{-11}
Age	—	—	—	-0.02	0.98 (0.96–1.01)	0.146	-0.01	0.99 (0.97–1.01)	0.330	-0.01	0.99 (0.97–1.01)	0.321
Sex	—	—	—	-0.03	0.97 (0.56–1.69)	0.922	-0.11	0.89 (0.51–1.57)	0.693	-0.13	0.88 (0.49–1.55)	0.650
BMI	—	—	—	1.00×10^{-3}	1.00 (0.93–1.08)	0.984	1.00×10^{-3}	1.00 (0.93–1.08)	0.985	0.01	1.01 (0.93–1.09)	0.809
Smoking	—	—	—	-0.30	0.74 (0.44–1.25)	0.258	-0.31	0.73 (0.43–1.25)	0.257	-0.37	0.69 (0.40–1.19)	0.182
Hypertension	—	—	—	—	—	—	0.28	1.33 (0.77–2.30)	0.312	0.33	1.39 (0.80–2.42)	0.248
Diabetes	—	—	—	—	—	—	0.46	1.58 (0.93–2.69)	0.089	0.41	1.50 (0.87–2.57)	0.142
CAD	—	—	—	—	—	—	-0.60	0.55 (0.27–1.13)	0.102	-0.61	0.54 (0.25–1.18)	0.122
Dyslipidemia	—	—	—	—	—	—	-0.39	0.68 (0.30–1.52)	0.345	-0.35	0.70 (0.31–1.60)	0.401
A-fib	—	—	—	—	—	—	—	—	—	-0.24	0.79 (0.18–3.38)	0.748
Thrombolysis	—	—	—	—	—	—	—	—	—	-2.28	0.10 (0.01–0.87)	0.037

Note: Model 1 was unadjusted and included HALP only. Model 2 was adjusted for age, sex, BMI, and smoking. Model 3 was further adjusted for hypertension, diabetes, CAD, and dyslipidemia. Model 4 was additionally adjusted for A-fib and thrombolysis. OR, odds ratio; CI, confidence interval; HALP, hemoglobin-albumin-lymphocyte-platelet; BMI, body mass index; CAD, coronary artery disease; A-fib, atrial fibrillation.

Supplementary Table 5. Heterogeneity analysis in the non-thrombectomy validation cohort

Variables	Model 1			Model 2			Model 3			Model 4		
	β	OR（95%CI）	*P*	β	OR（95%CI）	*P*	β	OR（95%CI）	*P*	β	OR（95%CI）	*P*-value
HALP	-0.05	0.95 (0.93–0.97)	5.501×10^{-5}	-0.05	0.96 (0.93–0.98)	3.157×10^{-4}	-0.05	0.95 (0.93–0.98)	2.695×10^{-4}	-0.05	0.95 (0.93–0.98)	3.678×10^{-4}
Age	—	—	—	0.03	1.03 (0.99–1.06)	0.102	0.03	1.03 (0.99–1.06)	0.135	0.03	1.03 (0.99–1.07)	0.094
Sex	—	—	—	-0.59	0.56 (0.24–1.32)	0.182	-0.82	0.44 (0.12–1.11)	0.081	-0.86	0.42 (0.16–1.10)	0.078
BMI	—	—	—	-0.10	0.90 (0.80–1.02)	0.092	-0.14	0.87 (0.76–0.99)	0.033	-0.14	0.87 (0.76–0.99)	0.042
Smoking	—	—	—	-0.48	0.62 (0.24–1.59)	0.322	-0.62	0.54 (0.20–1.46)	0.224	-0.79	0.46 (0.17–1.24)	0.125
Hypertension	—	—	—	—	—	—	0.51	1.66 (0.66–4.19)	0.286	0.51	1.67 (0.64–4.36)	0.296
Diabetes	—	—	—	—	—	—	0.53	1.71 (0.77–3.80)	0.191	0.51	1.67 (0.74–3.79)	0.221
CAD	—	—	—	—	—	—	-0.19	0.83 (0.30–2.28)	0.712	-0.05	0.95 (0.33–2.72)	0.921
Dyslipidemia	—	—	—	—	—	—	1.25	3.49 (1.16–10.48)	0.026	1.08	2.95 (0.97–8.96)	0.056
A-fib	—	—	—	—	—	—	—	—	—	-1.20	0.30 (0.03–3.26)	0.323
Thrombolysis	—	—	—	—	—	—	—	—	—	-1.51	0.22 (0.04–1.16)	0.074

Note: Model 1 was unadjusted and included HALP only. Model 2 was adjusted for age, sex, BMI, and smoking. Model 3 was further adjusted for hypertension, diabetes, CAD, and dyslipidemia. Model 4 was additionally adjusted for A-fib and thrombolysis. OR, odds ratio; CI, confidence interval; HALP, hemoglobin-albumin-lymphocyte-platelet; BMI, body mass index; CAD, coronary artery disease; A-fib, atrial fibrillation.

Note: β values are logistic regression coefficients. OR (95% CI) was calculated as exp(β ± 1.96×SE). P-values <0.001 are shown in scientific notation using ×10 superscript form

Supplementary Table 6. Multivariate logistic regression analysis of END risk factors

Variables	β	S.E	Z	*P*	OR (95%CI)
Intercept	0.12	0.85	0.14	0.892	1.12 (0.21-5.97)
Age	-0.01	0.01	-0.99	0.324	0.99(0.97-1.01)
Male	-0.03	0.27	-0.11	0.910	0.97(0.57-1.66)
Hypertension	0.25	0.26	0.97	0.333	1.29(0.77-2.15)
Diabetes	0.28	0.26	1.09	0.276	1.32(0.80-2.19)
Dyslipidemia	-0.48	0.40	-1.21	0.227	0.62(0.29-1.35)
LAA	1.60	0.28	5.81	<0.001	4.98(2.90-8.56)
NIHSS	0.04	0.02	2.30	0.021	1.04(1.01-1.08)
HALP	-0.04	0.01	-5.14	<0.001	0.96(0.95-0.98)
Smoking	-0.24	0.26	-0.91	0.361	0.79(0.47-1.32)

Note: OR, odds ratio; CI, confidence interval; LAA, large-artery atherosclerosis; NIHSS, National Institutes of Health Stroke Scale; HALP, hemoglobin-albumin-lymphocyte-platelet;

Supplementary Table 7. Indicators such as the AUC and sensitivity of the ROC curve of the nomogram model for predicting the risk of END

Dataset	AUC (95%CI)	Accuracy (95%CI)	Sensitivity (95%CI)	Specificity (95%CI)	Cut off
Train	0.78(0.74-0.83)	0.74(0.69-0.78)	0.66(0.58-0.74)	0.77(0.72-0.82)	0.392
Test	0.79(0.72-0.86)	0.71(0.64-0.77)	0.75(0.64-0.86)	0.69(0.61-0.77)	0.392

Note: AUC, area under the curve; ROC, receiver operating characteristic; END, early neurological deterioration.

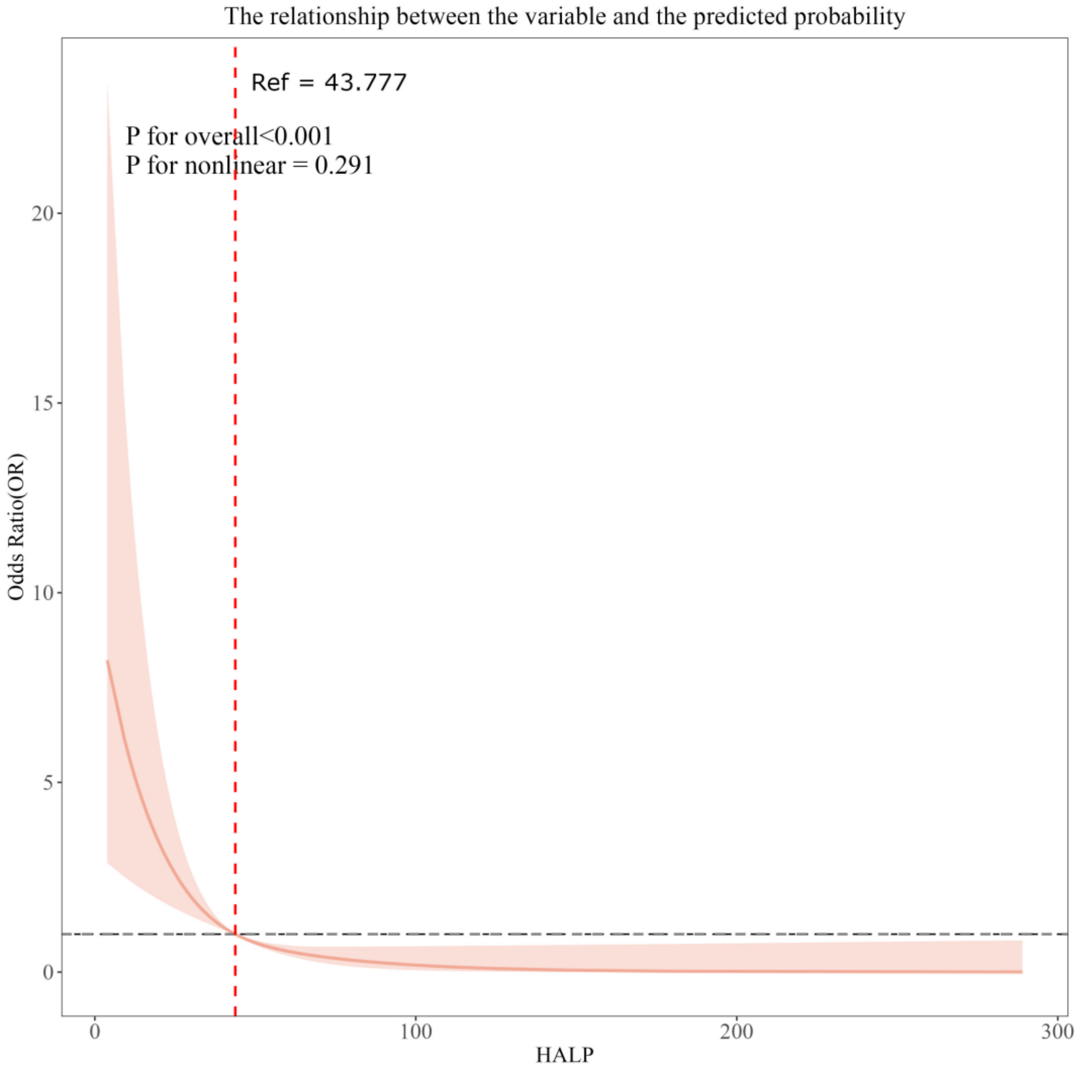

Supplementary Figure 1. Restricted cubic spline analysis of the association between HALP and END risk in the training cohort.

The restricted cubic spline analysis showed a significant overall association between HALP and END risk (*P* for overall < 0.001). The test for nonlinearity was not statistically significant (*P* for nonlinearity = 0.291), suggesting no clear evidence of a nonlinear association between HALP and END in the present dataset. Therefore, HALP was retained as a continuous variable in the logistic regression model. END, early neurological deterioration; HALP, hemoglobin-albumin-lymphocyte-platelet.

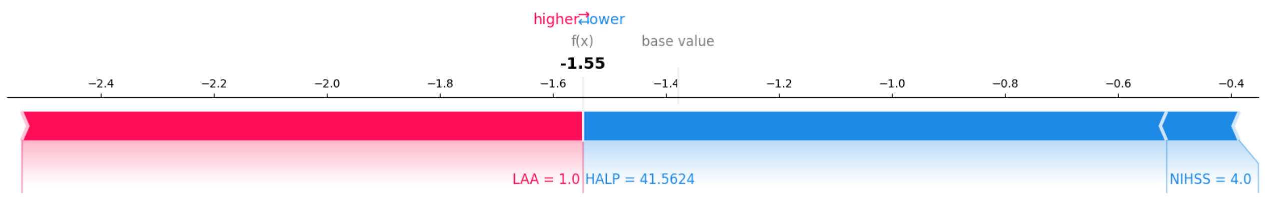

Supplementary Figure 2. SHAP force plot for individual-level interpretation of the combined model. The SHAP force plot illustrates how individual predictors shift the model output from the base value to the final prediction for one representative patient. Red bars indicate features that increase the model output, whereas blue bars indicate features that decrease the model output. In this example, LAA subtype (LAA = 1.0) contributed positively to the model output for END risk, while HALP (41.56) and NIHSS score (4.0) contributed negatively. The final model output was f(x) ≈ −1.55 on the log-odds scale, which was slightly lower than the base value, indicating that the negative contributions of HALP and NIHSS outweighed the positive contribution of LAA in this individual prediction. SHAP, SHapley additive explanations; END, early neurological deterioration; HALP, hemoglobin-albumin-lymphocyte-platelet; NIHSS, National Institutes of Health Stroke Scale; LAA, large-artery atherosclerosis.

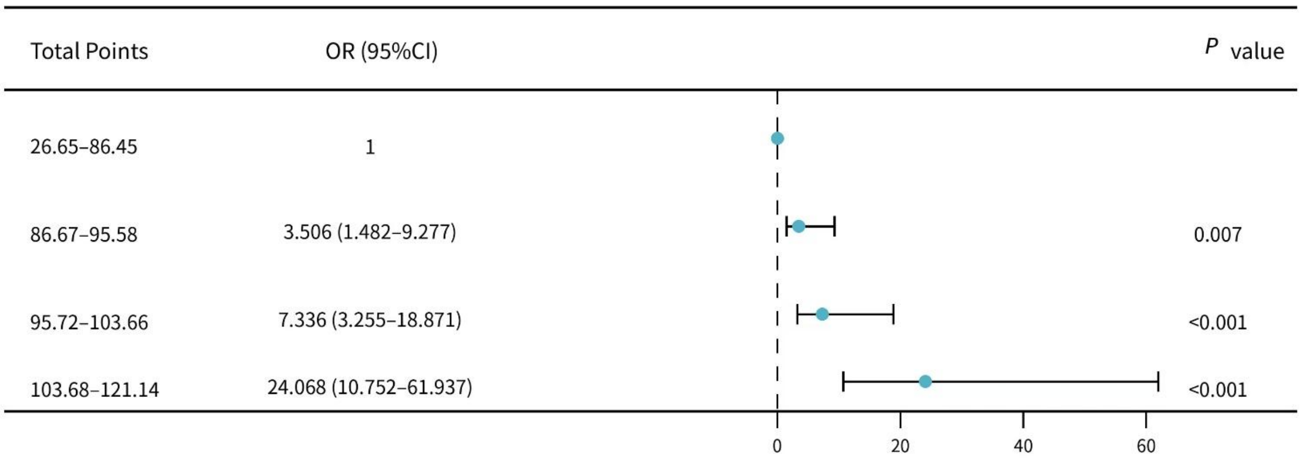

Supplementary Figure 3. Association between different total points intervals and END. The total points of the training cohort were divided into quartiles and incorporated into a logistic regression model to evaluate the risk of END across different risk groups. The risk of END increased with increasing total points. Compared with those in the lowest quartile, participants in the highest quartile (total points: 103.68-121.14) had a higher risk of END (OR = 24.07, 95% CI: 10.75-61.94). END, early neurological deterioration.
